# The Glycerole Compound as Remedy for Internal Hemorrhoids

**Published:** 1888-08

**Authors:** Q. A. Shuford

**Affiliations:** Tyler, Texas


### THE GLYCEROLE COMPOUND AS REMEDY FOR INTERNAL HEMORRHOIDS.

By Q. A. Shuford, M. D., Tyler, Texas.

For Daniel's Texas Medical Journal.

FOR aborting furuncle, (as yet I only have one case, but as that one is of such interest, and so typical of many others, I think that I am justified in reporting it, as the profession wish facts and remedies that can be depended on.)

Mr. John L——, of Tyler, has suffered with furuncles almost continuously for ten years, and for the last year they have grown rapidly worse, so much so that he has been disabled from ordinary business, frequently having from three to five large phlegmons at a time, on different parts of his person. His great suffering with pain and prostration from fever, together with the large amount of purulent matter in his system, clearly indicated septicæmia. Now, for the relief of this trouble, was the important question.

Mr. L—— has had the skill of the best physicians in this country all the while. Everything that science and art could suggest was brought to bear, both locally and constitutionally, upon his case. All failed, and as he was rapidly growing worse, I concluded to try this glycerole compound, or "pile fluid," and I am happy to say it had the desired effect—it acted like a charm.

The patient was directed to saturate a piece of absorbent cotton, and carefully apply it to the pimple, or pustule, and not let it spread so as to affect the surrounding parts. The inflammation was at once arrested, a scab would form, and when removed the core would follow. One or two more applications would be all that was necessary. Since adopting this remedy, the alteratives and tonics have had good effect, and the patient is now well.

While one case, as a general thing, does not prove much, yet, under certain circumstances, it may prove a great deal. This was a case of vitiated system, without any history of specific taint.

Dr. J. H. McBride, Denton, reports three cases treated successfully with glycerole compound. Two cases of alveola abscess, and one case of abscess of the antrum of Higmore. The fluid was injected into the cavity every day, and with proper drainage, the abscess healed rapidly.

All who have treated these troublesome abscesses, know how gladly they would receive a remedy that they could rely upon.

It is equally efficient in the treatment of carbuncle, ulcer, etc.

Since writing this report, some months ago, I have had a chance to try the remedy on several cases of boils, and it has stopped them in every instance.

I am very anxious for the profession to give it a fair trial. I shall try it in the first case of erysipelas that I have to treat, upon general principles.

[Dr. Shuford's formula is as follows:—ED.]

℞ Glycerole of boric acid.
Glycerole of salicylic acid, aa ℥ iv.
Pure carbolic acid, ℥ iii.

M. Rub thoroughly in a mortar.

